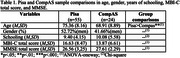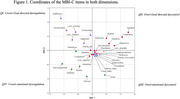# MBI‐C structure in MBI participants from the CompAS and Pisa cohort studies: A Multidimensional Scaling Approach

**DOI:** 10.1002/alz70857_099858

**Published:** 2025-12-24

**Authors:** Sabela C. Mallo, Camilla Elefante, Eulogio Real‐Deus, Giulio Emilio Brancati, Onésimo Juncos‐Rabadán, Giulio Perugi, Terezie Vohrádková, Zahinoor Ismail, Arturo X. Pereiro Rozas

**Affiliations:** ^1^ Instituto de Psicoloxía (IPsiUS), Universidade de Santiago de Compostela, Santiago de Compostela, Galicia, Spain; ^2^ Department of Clinical and Experimental Medicine ‐ Psychiatry Unit, University of Pisa, Pisa, Italy; ^3^ Department of Social Psychology and Methodology, University of Santiago de Compostela, Santiago de Compostela, Spain; ^4^ Departamento de Psicoloxía Evolutiva e da Educación, Universidade de Santiago de Compostela, Santiago de Compostela, Galicia, Spain; ^5^ Applied Cognitive Neuroscience and Psychogerontology group, Health Research Institute of Santiago de Compostela (IDIS), Santiago de Compostela, Spain; ^6^ Memory Clinic, Department of Neurology, Charles University, 2nd Faculty of Medicine and Motol University Hospital, Prague, Czech Republic; ^7^ Hotchkiss Brain Institute, University of Calgary, Calgary, AB, Canada

## Abstract

**Background:**

The Mild Behavioral Impairment Checklist (MBI‐C) is a 34‐question tool that assess neuropsychiatric symptoms (NPS) in individuals at risk of dementia. However, its foundational structure is still not well understood. Multidimensional Scaling (MDS) has some advantages that make it appropriate to analyze MBI‐C's structure:(1) provides orthogonal, normalized dimensions to explain items relationships; (2) is iterative, not analytic, requiring no prior assumptions about the data; (3) offers simpler solutions with fewer dimensions for a good fit. Thus, we aimed to analyze the underlying structure of the MBI‐C at baseline by using MDS in a sample of MBI participants from the CompAS and the Pisa cohort studies.

**Method:**

Participants from the CompAS and Pisa studies were recruited, respectively, at primary care health centers and psychogeriatric outpatient service. Pre‐dementia participants from the Pisa and the CompAS studies completed the MBI‐C and only those with met the MBI diagnosis criteria were considered (Pisa=55; CompAS=24). Regarding the MDS analyses, a two‐step bidimensional weighted dichotomous MDS was performed.

**Results:**

Study comparisons showed only significant age differences, being the participants from the Pisa study older than those from the CompAS. No significant differences were found in the other variables (see Table 1).

The MDS analyses showed optimal fit indices (stress‐II = .25; D.A.F. = .98).

Figure 1 shows the coordinates for the MBI‐C items in the bidimensional solution. Dimension I (horizontal) differentiate between internalizing vs externalizing symptoms. Dimension II (vertical) distinguishes between behavioral‐goal directed dyscontrol vs emotional dysregulation. Thus, these quadrants indicate symptoms of Covert‐Goal directed dysregulation (I, top left); Overt‐Goal directed dyscontrol (II, top right); Overt‐emotional dyscontrol (III, bottom right); and Covert‐emotional dysregulation (IV, bottom left).

**Conclusion:**

Results suggest two main criteria underline NPS included in the original scale: internalizing vs externalizing symptoms and behavioral‐goal directed dyscontrol vs emotional dysregulation. These two criteria seem to differentiate between four NPS states (Covert‐Goal directed dysregulation, Overt‐Goal directed dyscontrol, Overt‐emotional dyscontrol, and Covert‐emotional dysregulation), which could be useful in the determination of risk factors for predementia participants.